# A Meibomian Gland Concretion Presenting as a Palpable Eyelid Mass: A Rare Case Report

**DOI:** 10.1155/crop/5352956

**Published:** 2026-10-09

**Authors:** Daisuke Hasegawa, Yui Nishijima, Satoko Kujiraoka, Naoyuki Matsumoto, Tatsuya Mimura

**Affiliations:** ^1^ Department of Ophthalmology Tsurumi University School of Dental Medicine Yokohama Kanagawa Japan tsurumi-u.ac.jp; ^2^ Department of Ophthalmology Keio University School of Medicine Shinjuku-ku Tokyo Japan keio.ac.jp; ^3^ Department of Diagnostic Pathology Tsurumi University Dental Hospital Yokohama Kanagawa Japan; ^4^ Department of Pathology Tsurumi University School of Dental Medicine Yokohama Kanagawa Japan tsurumi-u.ac.jp

**Keywords:** chalazion mimic, eyelid mass, large concretion, meibomian gland concretion, meibomian gland obstruction

## Abstract

**Background:**

Meibomian gland obstruction is a condition in which lipid secretions accumulate within the meibomian gland duct because of blockage of the gland orifice, leading to inflammation and the formation of a nodular lesion. In most cases, whitish lipid material can be observed at the eyelid margin. However, the formation of a true concretion within the meibomian gland duct is uncommon. Furthermore, large or clinically apparent meibomian gland concretions presenting as a palpable eyelid mass appear to be uncommon. In this report, we describe a rare presentation in which multiple interconnected concretions formed a clinically visible and palpable eyelid‐margin mass.

**Case Presentation:**

A 64‐year‐old woman presented with a painful mass in the right lower eyelid that had gradually enlarged over the previous year. Slit‐lamp examination revealed dilation of the meibomian gland orifices and a whitish lesion measuring approximately 6 × 3 mm along the eyelid margin. The overlying eyelid skin was thinned, allowing the underlying mass to be partially visible. Under local anesthesia, three vertical incisions were made at the meibomian gland orifices, and multiple white concretions were removed. The concretions were found to be interconnected beneath the meibomian gland duct and were excised in fragments. Histopathological examination demonstrated degenerative material with prominent cholesterol clefts and minimal inflammatory cell infiltration, findings compatible with a concretion. Postoperatively, the eyelid contour improved and the patient′s pain resolved.

**Conclusions:**

This case demonstrates a rare presentation of multiple interconnected concretions forming a clinically visible and palpable eyelid‐margin mass. The combination of the clinical and operative findings, macroscopic appearance, and histopathological characteristics supported a diagnosis of meibomian gland concretion, although glandular or ductal tissue was not identified in the examined fragments.

## 1. Introduction

Meibomian gland obstruction is a common eyelid disorder characterized by blockage of the glandular orifices, resulting in retention of meibum within the ducts and subsequent eyelid inflammation or nodular lesions [1]. Various factors, including meibomian gland dysfunction, chronic inflammation, and altered meibum composition, have been implicated in the pathogenesis of this condition [1]. In some cases, obstructive material may accumulate within the meibomian gland ducts and form meibomian gland concretions. These lesions are typically small and are associated with the meibomian gland ducts or acini, sometimes following the course of the glands [2, 3]. The term “meibomian gland concretion” is used here to distinguish these gland‐associated deposits from conjunctival concretions, which are discrete deposits located within the subepithelial space of the palpebral or forniceal conjunctiva and may cause foreign‐body sensation when they erode through the overlying conjunctival epithelium [4, 5]. However, the development of large or clinically apparent meibomian gland concretions causing palpable eyelid masses is considered extremely rare.

Because typical concretions occur on the tarsal conjunctival side and are usually very small, they are seldom visible through the eyelid skin and rarely present as a palpable eyelid mass. When a relatively large lesion develops in the eyelid, clinicians more commonly suspect chalazion, sebaceous gland disorders, or eyelid tumors rather than meibomian gland concretion [6].

Furthermore, the clinical morphology of meibomian gland concretions has not been well characterized in cases where multiple concretions coalesce to form a larger mass within the eyelid [4, 7]. Such lesions may obstruct the meibomian gland duct and potentially lead to chronic inflammation, eyelid swelling, or pain [3, 5]. However, reports describing large meibomian gland concretions forming a palpable eyelid mass are extremely limited in the literature.

Here, we report a rare case of a large meibomian gland concretion presenting as a palpable mass in the lower eyelid. The lesion consisted of multiple interconnected concretions that required surgical removal through the meibomian gland orifices. This case highlights an unusual clinical presentation of meibomian gland concretion and emphasizes the importance of considering this entity in the differential diagnosis of eyelid masses.

## 2. Case Report

A 64‐year‐old woman presented with a painful mass in the right lower eyelid. She had first noticed a small nodule in the same location approximately 5 years earlier; however, because it was asymptomatic, she had not sought medical attention. During the subsequent several years, the lesion remained small and asymptomatic, with no apparent substantial change in size or symptoms. Approximately 1 year before presentation, however, the lesion began to gradually enlarge and became increasingly painful. One year before presentation, the lesion gradually enlarged and became painful, prompting referral to our department for further evaluation and treatment.

Her past medical history, medication history, and allergy history were unremarkable. At the initial examination, the best‐corrected visual acuity was 1.2 (decimal notation) in both eyes. Intraocular pressure was 16 mmHg in the right eye and 15 mmHg in the left eye. Anterior segment and fundus examinations revealed no remarkable abnormalities other than the eyelid lesion.

Slit‐lamp examination demonstrated dilation of the meibomian gland orifices in the right lower eyelid. A whitish subcutaneous mass measuring approximately 6 × 3 mm was observed along the eyelid margin. The overlying eyelid margin skin appeared thinned, allowing the underlying mass to be partially visible through the skin (Figure 1).

**Figure 1:**
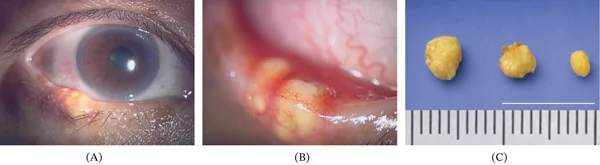
Slit‐lamp photographs of the right lower eyelid at the initial visit and the excised meibomian gland concretions. (A) Slit‐lamp image showing dilatation of the meibomian gland orifices along the eyelid margin. (B) A whitish lesion measuring approximately 6 × 3 mm is observed within the eyelid margin. Thinning of the overlying eyelid skin allows partial visualization of the underlying white mass. (C) Three excised meibomian gland concretions. Scale bar = 1.0 cm.

Based on these clinical findings, meibomian gland concretion or chronic chalazion was suspected, and surgical removal was performed under local anesthesia. During the procedure, three vertical incisions were made in the eyelid skin corresponding to the meibomian gland orifices to enlarge the openings. During the procedure, three small vertical incisions were made at the eyelid margin corresponding to the dilated meibomian gland orifices to enlarge the gland openings. The whitish, stone‐like material was then gently extracted through the corresponding meibomian gland orifices. The lesion was located immediately beneath the eyelid margin and appeared to extend along the course of the meibomian glands. The lesion was not adherent to the surrounding eyelid tissues and could be removed easily without extensive dissection. Several concretions were interconnected and formed a continuous stone‐like mass beneath the gland openings. The mass was divided into smaller fragments to facilitate extraction. Three superficial concretions measuring approximately 3 mm in diameter and several smaller concretions (< 1 mm) from the deeper portion were removed. No distinct capsule or membranous lining surrounding the lesion was identified, and no apparent residual material remained after extraction.

To facilitate extraction through the gland openings, the mass was divided into smaller fragments. Consequently, three superficial concretions measuring approximately 3 mm in diameter and several smaller concretions (< 1 mm) located in the deeper portion were removed.

Histopathological examination was performed on two of the larger specimens. The histopathological findings are shown in Figure 2. Macroscopically, the specimens were fragile, yellowish‐white concretions. Microscopically, the lesions consisted predominantly of degenerative material with extensive cholesterol cleft formation. Histopathological examination was performed on two of the larger fragmented specimens. Macroscopically, the specimens were fragile, yellowish‐white concretions. Microscopically, both specimens consisted predominantly of degenerative material with extensive cholesterol cleft formation and a small number of inflammatory cells, predominantly neutrophils. Spherical aggregate‐like structures were also observed; however, no special staining, microbiological culture, or other confirmatory testing for microorganisms was performed. Therefore, these structures were described only as bacterial aggregate‐like structures and were not interpreted as evidence of infection. No neoplastic cellular components were identified in the two examined specimens. Although glandular or ductal tissue was not identified in the examined fragments, the clinical and operative findings demonstrated that the concretions were located directly beneath the dilated meibomian gland orifices and could be extracted through the corresponding gland openings. Taken together, these findings supported the diagnosis of a large aggregated meibomian gland concretion.

**Figure 2:**
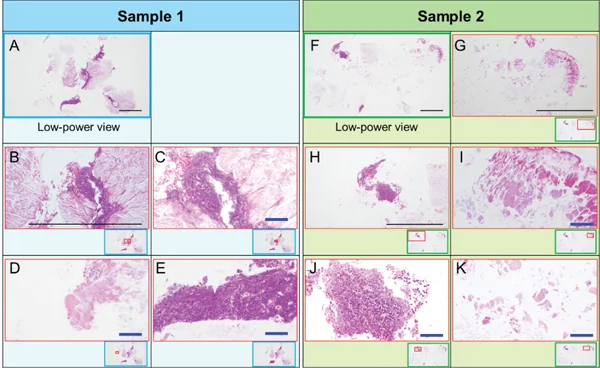
Histopathological findings of the excised meibomian gland concretions. (A) Low‐magnification view of Specimen 1 (×40). (B) Degenerative material with prominent cholesterol clefts and inflammatory cell infiltration, mainly neutrophils (×200). (C–E) Higher magnification views of a portion of Panel A, demonstrating degenerative material with inflammatory cell infiltration including neutrophils (×400). (F) Low‐magnification view of Specimen 2 (×40). (G,H) Higher magnification views of a portion of Panel F, showing degenerative material with neutrophil‐dominant inflammatory infiltration (×100). (I) Higher magnification view showing degenerative material and spherical bacterial aggregate‐like structures (×400). (J) Higher magnification view demonstrating degenerative material with inflammatory cell infiltration including neutrophils (×400). (K) Higher magnification view showing degenerative material and spherical bacterial aggregate‐like structures (×400). Black bars = 1.0 mm (A,B,F–H). Blue bars = 100 * μ*m (C–E,I–K).

Postoperatively, 1.5% levofloxacin ophthalmic solution and betamethasone sodium phosphate ophthalmic solution were administered to the right eye four times daily, and betamethasone valerate–fradiomycin sulfate ophthalmic ointment was applied twice daily. At the 1‐week postoperative follow‐up, the meibomian gland orifices had decreased in size, and the eyelid margin had become smooth and flattened (Figure 3). The patient also reported marked improvement in pain. Postoperatively, topical medication was administered for 2 weeks. At the most recent follow‐up, 8 months after surgery, no recurrence or other complications, including infection, were observed. A schematic illustration of the structure of the meibomian glands and the meibomian gland concretion observed in the present case is shown in Figure 4.

**Figure 3:**
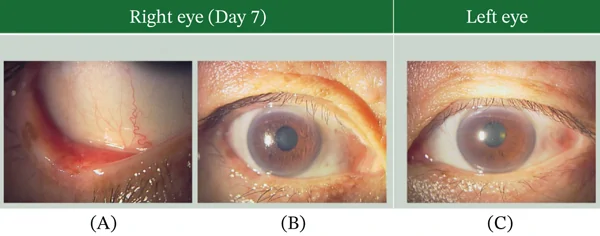
Slit‐lamp photographs obtained 1 week after surgery. (A) Right lower eyelid showing reduction of the meibomian gland orifices after removal of the concretions. (B) Slit‐lamp photograph of the right eye demonstrating a smooth and flattened lower eyelid margin following concretion removal. (C) Slit‐lamp photograph of the left eye. No apparent asymmetry of the eyelid contour is observed compared with the right eye.

**Figure 4:**
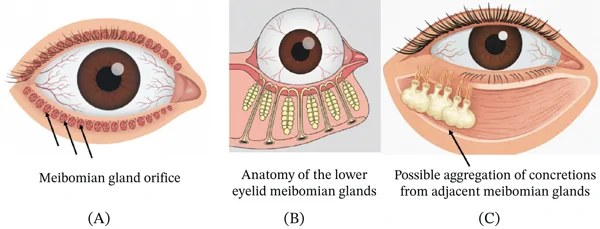
Schematic illustration of the meibomian gland structure and a hypothetical mechanism for the formation of a large aggregated concretion. (A) Meibomian gland orifice at the eyelid margin. (B) Normal anatomical structure of the meibomian glands in the lower eyelid. Multiple vertically oriented meibomian glands open onto the eyelid margin through individual glandular orifices. (C) Hypothetical model illustrating the possible aggregation of concretions associated with adjacent meibomian glands, which may result in an apparently continuous or clinically visible mass. The proposed mechanism is hypothetical and was not directly demonstrated by anatomical or histopathological examination in the present case.

## 3. Discussion

Meibomian gland concretions are uncommon lesions associated with the meibomian gland ducts or acini and are generally small in size [2, 3]. They may follow the anatomical course of the meibomian glands and can be identified in association with the glandular structures or their orifices [3]. This entity should be distinguished from conjunctival concretions, which are discrete yellowish‐white deposits that typically occur within the subepithelial space of the palpebral or forniceal conjunctiva. Conjunctival concretions are usually asymptomatic but may cause foreign‐body sensation or ocular irritation when they erode through the overlying conjunctival epithelium [4].

The anatomical location and operative findings also helped distinguish the lesion from other types of eyelid deposits. Conjunctival concretions typically arise beneath the palpebral conjunctival epithelium and are not directly associated with dilated meibomian gland orifices. In the present case, the whitish material was observed immediately beneath the dilated meibomian gland orifices and was directly extracted through these openings, with multiple concretions appearing interconnected beneath the gland openings. A calcified chalazion was considered less likely because the lesion consisted of multiple friable, yellowish‐white concretions rather than a discrete inflammatory granulomatous mass, and histopathological examination did not demonstrate a typical granulomatous inflammatory lesion or neoplastic component. Because the patient was 64 years old and the lesion had gradually enlarged over several years, an eyelid neoplasm was considered in the initial differential diagnosis. However, the whitish coloration, marked dilation of the meibomian gland orifices, and localization of the lesion beneath the eyelid margin favored a meibomian gland–associated lesion. Histopathological examination of the available specimens showed no neoplastic cellular components. Nevertheless, because the lesion was fragmented during extraction, only two specimens were examined, and glandular or ductal tissue was not identified—a neoplastic process elsewhere in the original lesion could not be completely excluded. The diagnosis was therefore based on the combination of the clinical and operative findings, macroscopic appearance, and histopathological characteristics of the examined specimens.

In contrast to the typical presentation, the present case demonstrated an unusually large meibomian gland concretion forming a palpable eyelid mass that was visible through the thinned eyelid skin. Such a presentation is extremely uncommon. Because meibomian gland concretions generally arise on the tarsal conjunctival side and remain small, they rarely present as a clinically apparent eyelid tumor‐like lesion. When a mass is identified within the eyelid, clinicians more frequently consider other conditions such as chalazion, sebaceous gland disorders, or benign and malignant eyelid tumors [6]. The present case should also be distinguished from our previously published case of meibomian gland infarction presenting as an eyelid tumor‐like lesion [6]. Although both cases involved an eyelid mass associated with meibomian gland abnormalities and were reported by the same authors, they represent separate patients and distinct clinical and pathological presentations. In the previous case, the lesion was diagnosed as meibomian gland infarction, whereas the present case consisted of multiple interconnected, yellowish‐white concretions located beneath dilated meibomian gland orifices and removable through the corresponding gland openings. Histopathologically, the present specimens consisted predominantly of degenerative material with prominent cholesterol clefts and limited inflammatory cell infiltration, without neoplastic cellular components. Thus, the clinical, operative, and pathological findings of the present case differed from those of the previously reported meibomian gland infarction. Taken together, these findings supported a diagnosis of aggregated meibomian gland concretion in the present patient.

Another notable feature of this case was that multiple concretions appeared to be interconnected and formed a larger aggregated mass beneath the meibomian gland orifices. The precise mechanism underlying the formation of such a large aggregated lesion remains unclear. Chronic obstruction of the meibomian gland ducts and stagnation of meibum may contribute to the accumulation of keratinized debris and lipid material and subsequent enlargement of individual concretions. Alternatively, concretions arising from adjacent meibomian glands may become aggregated or appear continuous as the lesion enlarges. However, no direct anatomical or histopathological evidence of interglandular septal destruction was obtained in the present case. Therefore, the mechanism illustrated in Figure 4 should be regarded as a hypothetical model rather than a demonstrated sequence of lesion formation. Chronic inflammation of the eyelid margin or meibomian gland dysfunction has also been suggested to contribute to the development of concretions [2, 5].

Because the patient was 64 years old and the eyelid lesion had gradually enlarged over several years, an eyelid neoplasm was considered in the clinical differential diagnosis. The lesion was carefully evaluated for features that could suggest a neoplastic process, including its progressive enlargement, palpable mass formation, and abnormal appearance of the eyelid margin. However, the whitish coloration, marked dilation of the meibomian gland orifices, and the apparent localization of the lesion beneath the eyelid margin also suggested a meibomian gland–associated lesion.

The clinical and pathological features of the present case differ from those of several previously described eyelid and ocular surface concretions. Meibomian gland concretions are recognized manifestations of meibomian gland disease and are generally small deposits associated with the glandular ducts. They may follow the course of the meibomian glands and are often identified on examination of the tarsal plate or gland orifices. In contrast, in the present case, multiple concretions were located immediately beneath dilated meibomian gland orifices and appeared interconnected, forming a clinically visible and palpable mass along the eyelid margin. The clinically visible lesion measured approximately 6 × 3 mm; however, the lesion was fragmented during extraction, and the individual removed concretions measured approximately 3 mm or less. Conjunctival concretions are generally small yellow‐white deposits located within the palpebral or forniceal conjunctiva and are often asymptomatic or associated with foreign‐body sensation when they protrude through the conjunctival epithelium. The lesion in the present case was not primarily located on the conjunctival surface but was situated beneath the eyelid margin and was extracted through dilated meibomian gland orifices. Histopathologically, the specimens predominantly consisted of degenerative material with prominent cholesterol clefts and limited inflammatory cell infiltration, without neoplastic components. Calcified eyelid lesions and chalazia represent additional differential diagnoses. In contrast to lesions characterized by mineralization or a granulomatous inflammatory, the specimens in the present case did not show a typical calcified or granulomatous lesion. Thus, the distinctive feature of the present case was not simply the absolute size of a single concretion, but the clinical presentation of multiple interconnected meibomian gland concretions forming a palpable eyelid‐margin mass.

Histopathological examination in the present case revealed degenerative material with prominent cholesterol clefts and limited inflammatory cell infiltration, predominantly consisting of neutrophils. These findings were compatible with the appearance of a meibomian gland concretion. Spherical aggregate‐like structures were also observed; however, because no specific microbiological staining or other confirmatory testing was performed, their nature could not be established and they were not interpreted as evidence of bacterial infection. Importantly, no neoplastic cellular components were identified in the two examined fragments. Because the lesion was fragmented during extraction, however, these findings should be interpreted with the limitation that only a portion of the original lesion was available for histopathological examination.

Clinically, large concretions may cause eyelid discomfort, pain, or localized swelling, particularly when they obstruct the meibomian gland orifice or induce secondary inflammation. Surgical removal is generally effective in relieving symptoms and restoring the patency of the meibomian gland duct. In the present case, multiple incisions along the meibomian gland orifices allowed successful removal of the aggregated concretions, resulting in improvement of both the eyelid contour and the patient′s symptoms.

This case highlights an unusual clinical presentation of meibomian gland concretion manifesting as a relatively large eyelid mass. Ophthalmologists should therefore consider large meibomian gland concretion in the differential diagnosis of eyelid masses, particularly when whitish material is visible near the meibomian gland orifices or when the lesion is associated with chronic eyelid margin changes.

Several limitations should be acknowledged. First, this report describes a single case, and the incidence and pathogenesis of large meibomian gland concretions remain unclear. Second, the long‐term recurrence rate after removal was not evaluated over an extended follow‐up period. Further accumulation of similar cases and clinical studies is required to clarify the mechanisms underlying the formation of large concretions and their optimal management.

## Author Contributions

All authors contributed to the conception and design of the study. T.M. collected and analyzed the data and drafted the manuscript. D.H. and Y.N. provided critical revisions of the manuscript. S.K. and N.M. performed histological processing, staining, microscopic imaging, and pathological evaluation of patient specimens. N.M. reviewed and edited the manuscript and managed project administration. All authors commented on previous versions of the manuscript.

## Funding

No funding was received for this manuscript.

## Disclosure

All authors approved the final version of the manuscript.

## Ethics Statement

This case report was conducted in accordance with the ethical principles of the Declaration of Helsinki (World Medical Association, 2013). Institutional ethics committee approval was not required for this case report according to the institutional policy for case reports. Written informed consent for publication of the clinical information and images was obtained from the patient.

## Consent

Written informed consent was obtained from the participant for publication of the case details and accompanying clinical photographs in an academic publication.

## Conflicts of Interest

The authors declare no conflicts of interest.

## Data Availability

Data sharing is not applicable to this article as no datasets were generated or analyzed during the current study.
